# Triple-Marker Screening of 20 Terpenes Identifies Candidate Modulators of Senescence-Associated Phenotypes in D-Galactose-Treated Endothelial Cells

**DOI:** 10.3390/molecules31101614

**Published:** 2026-05-11

**Authors:** Arthur José Pontes Oliveira de Almeida, Larisse Virgolino da Silva Pontes, Javanyr Frederico de Souza Júnior, Evyllen Myllena Cardoso Soares, Adhonias Correia dos Santos, Tays Amanda Felisberto Gonçalves, Mathania Silva de Almeida Feitosa, Robson Cavalcante Veras, Jose Maria Barbosa Filho, Lisa A. Lesniewski, Anthony J. Donato, Isac Almeida de Medeiros

**Affiliations:** 1 Post-Graduate Program in Development and Technological Innovation in Medicines, Health Sciences Center, Federal University of Paraíba, Campus I, João Pessoa 58071-970, PB, Brazil; arthur.almeida@utah.edu; 2 Department of Pharmaceutical Sciences, Health Sciences Center, Federal University of Paraíba, Cidade Universitária—Campus I, João Pessoa 58051-970, PB, Brazil; larisse.pontes@utah.edu (L.V.d.S.P.); evyllen.cardoso@academico.ufpb.br (E.M.C.S.); taysamanda@ltf.ufpb.br (T.A.F.G.); mathaniarez@ltf.ufpb.br (M.S.d.A.F.); robsonveras@ccs.ufpb.br (R.C.V.); jbarbosa@ltf.ufpb.br (J.M.B.F.); 3 Nora Eccles Harrison Cardiovascular Research and Training Institute, University of Utah, Salt Lake City, UT 84112, USA; lisa.lesniewski@utah.edu (L.A.L.); tony.donato@hsc.utah.edu (A.J.D.); 4 Post-Graduate Program in Bioactive Natural and Synthetic Products, Health Sciences Center, Federal University of Paraíba, Campus I, João Pessoa, 58051-900, PB, Brazil; javanyrjunior@ltf.ufpb.br (J.F.d.S.J.); adhonias.correia@academico.ufpb.br (A.C.d.S.); 5 Department of Nutrition and Integrative Physiology, University of Utah, Salt Lake City, UT 84112, USA; 6 Department of Internal Medicine, Division of Geriatrics, University of Utah, Salt Lake City, UT 84112, USA; 7 Veterans Affairs Medical Center–Salt Lake City, Geriatric Research Education and Clinical Center, Salt Lake City, UT 84148, USA; 8 Department of Biochemistry, University of Utah, Salt Lake City, UT 84112, USA; 9 Department of Molecular Biology, University of Utah, Salt Lake City, UT 84112, USA

**Keywords:** aging, antioxidants, endothelial cells, essential oils, senescence, terpenes

## Abstract

Aging is a major risk factor for chronic diseases, including cardiovascular diseases. Endothelial cells (ECs) are particularly vulnerable to age-related stress, and endothelial senescence contributes to vascular aging. In the present study, we applied a triple-screening strategy to identify terpenes that modulate senescence-associated phenotypes in endothelial cells. Rat aortic endothelial cells (RAECs) were exposed to D-galactose to induce a senescence-associated phenotype, and 20 g/L was selected for subsequent experiments. Subsequently, twenty terpenes were tested at four concentrations (10^−9^, 10^−8^, 10^−7^, and 10^−6^ mol/L) using three phenotypic readouts: senescence-associated β-galactosidase (SA-β-Gal) activity, cell viability, and oxidative stress. The screening identified multiple terpenes that reduced SA-β-Gal activity and oxidative stress, while five compounds improved cell viability under D-galactose conditions. Among them, citral, terpinolene, and farnesol were the only compounds that showed concordant beneficial effects across the three screening endpoints at the same concentration. In addition, 1*R*-(-)-myrtenol and *trans*-caryophyllene showed lower SA-β-Gal activity and preferentially reduced cell viability in D-galactose-treated cells, warranting follow-up studies to determine whether they exhibit senolytic activity. Overall, these findings identify terpene candidates that modulate senescence-associated phenotypes in ECs and support further mechanistic studies to define their senescence-related actions and potential relevance for natural-product-based strategies targeting EC dysfunction.

## 1. Introduction

Aging is the major risk factor for a broad range of chronic diseases, particularly cardiovascular diseases (CVDs), which remain the leading cause of morbidity and mortality in older adults [1,2,3]. This increased susceptibility results from molecular, cellular, and physiological changes during aging, highlighting endothelial dysfunction, an early event in cardiovascular aging [4,5]. With aging, endothelial cells become increasingly vulnerable to oxidative, inflammatory, and metabolic stress, leading to functional impairment and contributing to the development of vascular disease [3,4,5]. Therefore, identifying compounds that attenuate endothelial dysfunction is of considerable interest for strategies aimed at extending healthspan and reducing age-related cardiovascular burden.

Cellular senescence has emerged as a hallmark of aging [6]. Senescent cells (SnCs) accumulate with age, resist apoptosis, acquire a senescence-associated secretory phenotype (SASP), and exhibit elevated ROS levels as part of their metabolic reprogramming, which amplifies inflammation and tissue dysfunction [7,8,9,10,11,12]. In endothelial cells, senescence has been linked to impaired nitric oxide bioavailability, increased oxidative stress, pro-inflammatory signaling, and reduced regenerative capacity, all of which contribute to vascular aging [12,13]. Therefore, RAECs were selected as a relevant vascular endothelial model for identifying candidate compounds in a context directly related to endothelial dysfunction and vascular aging.

Several experimental models have been developed to study cellular senescence in vitro and in vivo, including replicative senescence driven by telomere attrition, DNA damage-induced senescence, oncogene-induced senescence, and senescence associated with metabolic stress [14,15,16,17,18]. These distinct models are valuable because they capture different upstream triggers and may generate partially different senescence phenotypes depending on the cell type and biological context, including the endothelium [17,18,19]. Among these, D-galactose has been widely used as an experimental model of accelerated aging [20,21]. D-galactose models are characterized by increased SA-β-Galactosidase (SA-β-Gal) activity, the most commonly used indicator of senescence [22]. Moreover, the D-galactose senescence model shows elevated levels of p16, p21, and p53, as well as elevated DNA damage (e.g., γ-H2AX) [21,23]. Additionally, among the processes linked to D-galactose-induced aging are the production of advanced glycation end products and the accumulation of ROS in cells [20,21,24].

Therapeutic strategies targeting SnCs, collectively known as senotherapeutics, include senolytics, which eliminate SnCs, and senomorphics, which suppress their harmful pro-aging phenotype [25,26,27]. While pre-clinical models support the benefits of senotherapeutics, including improvements in cardiovascular and metabolic function, physical capacity, reduced frailty, and increased survival [10,11,28,29,30,31], in clinical trials, some candidates exhibit toxicity (e.g., thrombocytopenia) and show cell- or tissue-specificity [32,33]. Therefore, the development of therapies that aim to reduce the deleterious consequences of senescence burden is necessary for the clinic [34,35].

Essential oils are natural products extracted from various aromatic plants and exhibit various biological properties [36]. These effects are attributed mainly to the terpenes, the major chemical components of these oils. Terpenes are bioactive metabolites with antioxidant, anti-inflammatory, and anticancer effects [36,37,38,39]. In addition, terpenes are associated with cardiovascular benefits, including improvement in endothelial function [40,41,42,43], but their role as senotherapeutic or geroprotective agents remains underexplored [44]. Thus, the senotherapeutic potential of terpenes not only opens new avenues for research but also brings hope for effective aging interventions.

Therefore, the present study aimed to identify candidate modulators of senescence-associated phenotypes through a triple-screening approach in endothelial cells exposed to D-galactose. To accomplish this, we used a triple-screening strategy based on three complementary phenotypic readouts: senescence-associated SA-β-Gal activity, cell viability, and oxidative stress.

## 2. Results

### 2.1. Adjustment of the D-Galactose Senescence Model in RAECs

**Figure 1:**
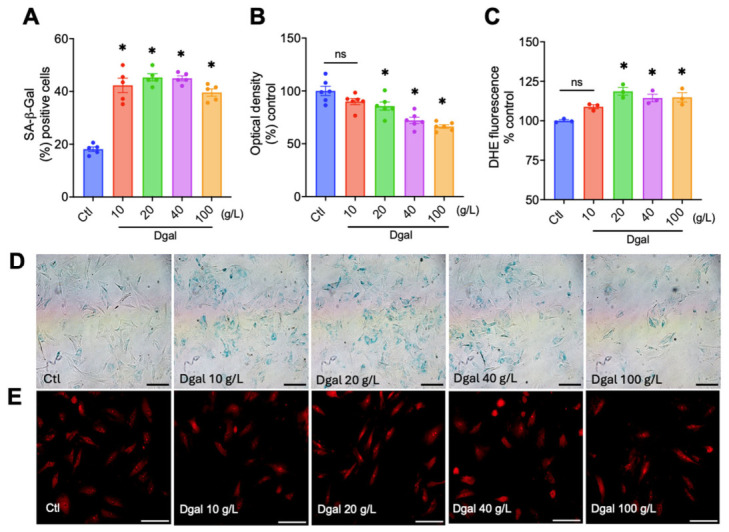
Optimization of the D-galactose-induced senescence-associated phenotype in RAECs. Quantification of (**A**) SA-β-Gal activity (*n* = 5), (**B**) cell viability (*n* = 6), and (**C**) oxidative stress (*n* = 3) after 48 h of exposure to increasing concentrations of D-galactose (10, 20, 40, and 100 g/L) in RAECs. (**D**) Representative brightfield images of SA-β-Gal staining. (**E**) Representative fluorescence images of dihydroethidium (DHE) as an indicator of oxidative stress. Data are expressed as mean ± SEM. Statistical analysis was performed using one-way ANOVA followed by Tukey’s post hoc test. ns = not significant. * *p* < 0.05 vs. Ctl. Scale bar = 100 µm.

D-galactose increased the number of senescent cells in RAECs after 48 h of exposure. The results were expressed as a percentage of senescent cells per total number of cells counted, with the Ctl group (23.9 ± 1.4). In this assay, the D-galactose 10 g/L (42.2 ± 2.7; *p* < 0.0001), D-galactose 20 g/L (45.3 ± 1.3; *p* < 0.0001), D-galactose 40 g/L (44.9 ± 0.9; *p* < 0.0001) and D-galactose 100 g/L (39.5 ± 1.3; *p* < 0.0001) groups had a significant increase in the number of senescent cells (Figure 1A). For the MTT assay, data were expressed as a percentage and normalized to the Ctl group (100 ± 4.4). In this assay, the D-galactose 10 g/L group (89.9 ± 1.6; *p* = 0.2291) showed no significant difference from the Ctl group. However, the D-galactose 20 g/L (85.6 ± 3.8; *p* = 0.0375), D-galactose 40 g/L (72.1 ± 2.9; *p* < 0.0001), and D-galactose 100 g/L (66.3 ± 1.6; *p* < 0.0001) groups showed a reduction in cellular viability (Figure 1B). Moreover, for the DHE assay, results were expressed as a percentage and normalized to the Ctl group (100 ± 1.4). In this assay, the D-galactose 10 g/L group (106.2 ± 1.9; *p* = 0.0842) showed no significant difference from the Ctl group. However, the D-galactose 20 g/L (116.6 ± 2.8; *p* = 0.0008), D-galactose 40 g/L (109.7 ± 1.2; *p* = 0.041), and D-galactose 100 g/L (113.7 ± 1.8; *p* = 0.0041) groups had increased oxidative stress (Figure 1C).

Accordingly, the 20 g/L concentration of D-galactose was used in further experiments as a model of endothelial senescence due to (a) increased SA-β-Gal activity, (b) increased superoxide anion levels, and (c) reduced cell viability.

### 2.2. Evaluation of SA-β-Gal Activity of Terpenes in the D-Galactose Senescence Model in RAECs

The screening of 20 terpenes to evaluate the activity of the lysosomal enzyme β-galactosidase associated with senescence revealed 18 promising terpenes in reducing the number of senescent cells compared to the D-galactose group (Table 1). Thus, the terpenes with significant improvement in relation to the D-galactose group and their respective concentrations were the following: geraniol, citral, linalool, β-citronellol, citronellal, α-terpineol, carvacrol, thymol, ρ-cymene, terpinolene, (-)-terpinen-4-ol, (+)-α-pinene, (-)-borneol, 1*R*-(-)-myrtenol, farnesol, nerolidol, α-bisabolol, and *trans*-caryophyllene.

### 2.3. Evaluation of Cell Viability Activity of Terpenes in a Basal Condition in RAECs

Exposure of 20 terpenes under basal conditions revealed five that reduced cell viability compared with the control group (Table 2). Thus, the terpenes capable of reducing cell viability under basal conditions were the following: citral, linalyl acetate, (-)-carveol, (-)-terpinen-4-ol, and (+)-α-pinene.

### 2.4. Evaluation of Cell Viability of Terpenes in the D-Galactose Senescence Model in RAECs

Screening 20 terpenes to assess cell viability revealed five promising terpenes that improved cell viability compared to the D-galactose group (Table 3). Thus, the terpenes with significant improvement were the following: geraniol, citral, α-terpineol, terpinolene, and farnesol. In contrast, two terpenes reduced cell viability beyond the levels of the D-galactose group: 1*R*-(-)-myrtenol and *trans*-caryophyllene.

### 2.5. Evaluation of Superoxide Anion Levels of Terpenes in the D-Galactose Senescence Model in RAECs

Twenty terpenes were screened for superoxide anion levels, and 18 of them were found to be promising antioxidants (Table 4). Thus, the terpenes with significant improvement in relation to the D-galactose group and their respective concentrations were the following: geraniol, citral, linalool, linalyl acetate, β-citronellol, citronellal, α-terpineol, carvacrol, thymol, terpinolene, carveol, (-)-terpinen-4-ol, (+)-α-pinene, (-)-borneol, farnesol, nerolidol, α-bisabolol, and *trans*-caryophyllene.

## 3. Discussion

The main finding of the present study is that several terpenes modulated senescence-associated phenotypes in endothelial cells. Cellular senescence has emerged as an important contributor to vascular aging and age-related dysfunction, and strategies targeting senescent cells or their deleterious phenotypes have attracted increasing attention. In this context, we used D-galactose as an established experimental approach to induce a senescence-associated phenotype and selected the 20 g/L concentration because it increased SA-β-Gal activity and oxidative stress while reducing cell viability. Thus, the present work was designed as a screening study to identify candidate terpenes with favorable activity across these three endpoints.

Terpenes are the major constituents of essential oils and often exhibit antioxidant and anti-inflammatory activities, thereby improving cell function and preventing senescence [36,39]. Here, we evaluated 20 terpenes with diverse chemical structures, including acyclic, monocyclic, and bicyclic monoterpenes and sesquiterpenes. However, despite the therapeutic potential of terpenes, they are rarely used as geroprotectors, and their ability to modulate senescence-associated phenotypes remains underexplored. Using the three selected screening readouts, we identified 18 terpenes that reduced SA-β-Gal activity, 18 that exhibited antioxidant effects, and 5 that enhanced cell viability (geraniol, citral, α-terpineol, terpinolene, and farnesol) in the D-galactose-induced senescence-like model. Furthermore, we evaluated the effects of 20 terpenes on cell viability under basal conditions to determine whether any had potentially unfavorable effects at these concentrations. We identified five terpenes that reduced cell viability under basal conditions (citral, linalyl acetate, (-)-carveol, (-)-terpinen-4-ol, and (+)-α-pinene), suggesting possible toxicity under these conditions. Moreover, 1*R*-(-)-myrtenol and *trans*-caryophyllene reduced SA-β-Gal activity and cell viability in the D-galactose-induced senescence-like model, while maintaining cell viability under basal conditions, a pattern warranting direct follow-up studies to determine whether these compounds exhibit senolytic activity.

These findings are broadly consistent with the previous literature, which indicates that terpenes can exert vasculoprotective and endothelial-supportive effects, particularly through antioxidant and redox-related mechanisms [45]. A systematic review and meta-analysis reported cardiovascular benefits for monoterpenes and their derivatives across preclinical studies [39]. In addition, carvacrol has been shown to improve vascular function in hypertensive animals and to attenuate D-galactose-induced aging-associated erectile dysfunction, effects associated with improved endothelial function and reduced oxidative stress [40,41]. Likewise, α-terpineol has been linked to vascular actions involving the nitric oxide-cGMP pathway [43]. Geraniol improved endothelial function in high-fat-diet-fed mice by inhibiting NOX2-derived oxidative stress [46], whereas citral protected human endothelial cells against hydrogen peroxide-induced oxidative injury [47]. Although these prior studies did not directly evaluate endothelial senescence, they support the biological plausibility of the beneficial effects observed in our screening approach.

Geraniol, citral, α-terpineol, terpinolene, and farnesol improved cell viability in the D-galactose-induced senescence-like model and did not reduce viability under basal conditions. Citral showed a narrow effective range for cell viability. Citral at 1 nM improved cell viability in the presence of D-galactose but not in basal conditions, and reduced SA-β-Gal activity and superoxide anion levels. However, citral lost its benefits at 10, 100, and 1000 nM. Additionally, geraniol and α-terpineol showed beneficial effects across multiple readouts; however, at different concentrations. Finally, citral 1 nM, terpinolene 100 and 1000 nM, and farnesol 100 nM showed triple efficacy in reducing SA-β-Gal activity, diminishing ROS levels, and improving cell viability. Under basal conditions, these three terpenes did not alter cell viability at their effective concentrations, suggesting that they may, at least in part, ameliorate senescence-associated phenotypes by reducing cellular stress.

Importantly, the present study differs from most previous reports because it was designed as a phenotype-based screen centered on senescence-associated readouts rather than on isolated vascular reactivity or anti-inflammatory endpoints. In this sense, our data extend the literature by suggesting that citral, terpinolene, and farnesol merit follow-up in mechanistic studies of endothelial senescence. This interpretation is also aligned with the broader proposal that terpenoids may act as geroprotective molecules [44], although direct validation of senescence pathways, such as p16, p21, and SASP markers, remains necessary.

Overall, the present findings position terpenes as relevant natural product candidates for future studies on vascular aging. By applying a simple phenotype-based screening strategy, this study provides an initial framework for prioritizing compounds for mechanistic evaluation, including validation in additional senescence models and characterization of cell-cycle arrest, DNA damage, and senescence-associated secretory phenotype pathways.

## 4. Materials and Methods

### 4.1. Animals

Eight-week-old male Wistar rats were purchased from the animal production unit of the Instituto de Pesquisa em Farmacos e Medicamentos (IPeFarM) of the Universidade Federal da Paraíba (UFPB), João Pessoa, PB, Brazil. The experimental animals were housed under conditions at 22 ± 1 °C with 12 h light–dark cycles and free access to water and food (Nuvilab CR-1, Quimtia^®^, Colombo, PR, Brazil). Animals were monitored daily. All animal procedures were conducted in accordance with the Health Guide for the Care and Use of Laboratory Animals, submitted and previously approved by the UFPB animal ethics committee (Ethic-approval number: 6267280422).

### 4.2. Isolation and Cell Culture

Primary rat aortic endothelial cells (RAECs) were isolated from rats (8–9 weeks old) as previously described [48]. The RAECs were cultured in DMEM (Dulbecco’s modified Eagle’s medium) low glucose (Sigma-Aldrich, Saint Louis, MO, USA), 10% FBS, no phenol red, L-Glutamine 4 mM (Sigma-Aldrich, Saint Louis, MO, USA), penicillin (100 units/mL) and streptomycin (100 µg/mL) (Sigma-Aldrich, Saint Louis, MO, USA), heparin 100 Units/mL (Hepamax-S®, Blau Farmacêutica S.A., Cotia, SP, Brazil), and maintained at 37 °C in a humidified incubator with 5% CO_2_. The culture medium was replaced every 2–3 days, and at 80% confluence, cells were split at a 1/3 ratio. Passages 2–3 were used for experiments.

### 4.3. Drugs

D-(+)-galactose (purity ≥ 98%), Kolliphor^®^ EL, dimethyl sulfoxide (DMSO), 5-bromo-4-chloro-3-indolyl β-D-galactopyranoside (x-gal), fluorescence mounting medium (DAKO^®^), glutaraldehyde, dihydroethidium (DHE), Dulbecco’s modified Eagle’s medium (DMEM), trypsin, penicillin and streptomycin were obtained from Sigma-Aldrich (Saint Louis, MO, USA). Ketamine and xylazine were acquired from Syntec (Santana de Parnaíba, SP, Brazil). Heparin (Hepamax-S®, Blau Farmacêutica S.A., Cotia, SP, Brazil); formaldehyde 10% from MedQuímica Indústria Farmacêutica Ltda (Juiz de Fora, MG, Brazil). Fetal bovine serum (FBS) was obtained from Induslab (Londrina, PR, Brazil); Professor Jose Maria Barbosa Filho kindly provided the terpenes. The terpenes were solubilized in DMSO and diluted daily in PBS to prepare the desired concentrations. DMSO was used as a vehicle.

**Figure 2:**
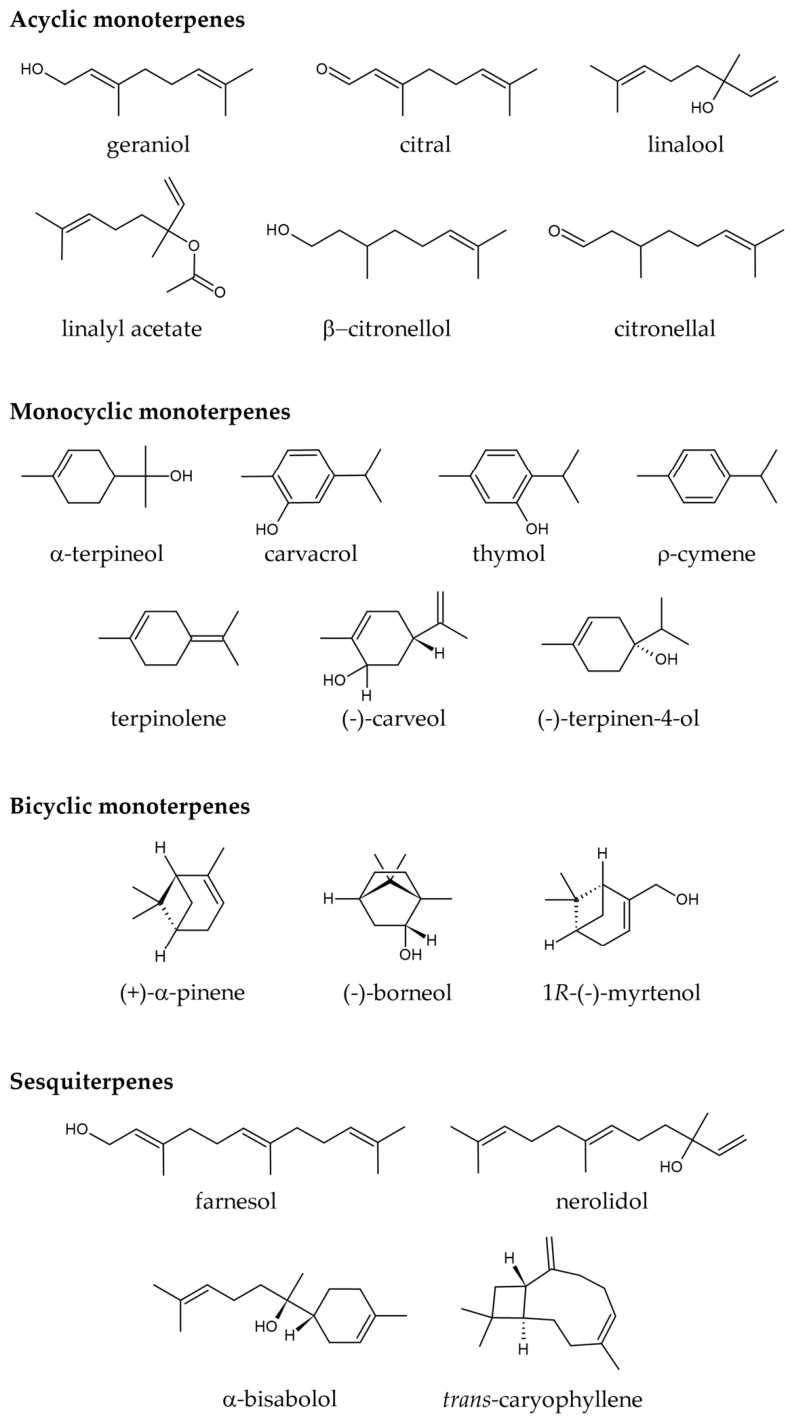
Chemical structures of terpenes used in the screening. The 20 terpenes evaluated in this study were selected to provide a chemically diverse initial screening panel representing major terpene subclasses commonly found as bioactive constituents of essential oils and are grouped as acyclic monoterpenes, monocyclic monoterpenes, bicyclic monoterpenes, and sesquiterpenes.

The 20 terpenes evaluated in this study were selected to provide a chemically diverse initial screening panel representing major terpene subclasses commonly found as bioactive constituents of essential oils. Based on structural similarity, they were grouped as follows: (1) acyclic monoterpenes (linalool, geraniol, citronellal, β-citronellol, citral, and linalyl acetate); (2) monocyclic monoterpenes (α-terpineol, carvacrol, thymol, ρ-cymene, (-)-carveol, terpinolene, and (-)-terpinen-4-ol); (3) bicyclic monoterpenes ((+)-α-pinene, (-)-borneol, and 1*R*-(-)-myrtenol); and (4) sesquiterpenes (farnesol, nerolidol, α-bisabolol, and *trans*-caryophyllene) (Figure 2).

### 4.4. Experimental Design

Prior to the experiment, RAECs were seeded at 2 × 10^4^ cells/well for 24 h. After this period, the cell culture medium was replaced by a medium containing D-galactose (10–100 g/L) for 48 h at 37 °C in a humidified incubator with 5% CO_2_ to select the desired concentration. The timeline and concentration range for D-Galactose were selected based on previous studies [21,49]. Then, the D-galactose-adjusted concentration was incubated with terpene concentrations (10^−9^, 10^−8^, 10^−7^, 10^−6^ M) for 48 h at 37 °C in a humidified incubator with 5% CO_2_. After cellular treatment, cells were washed 3× with PBS and were ready for pharmacological screening to assess senescence, oxidative stress, and cell viability.

### 4.5. β-Galactosidase Measurements

Senescence-associated β-galactosidase-positive cells were identified as previously described [22]. Briefly, after 48 h of treatment, cells were washed with phosphate-buffered saline (PBS) and then fixed with a solution of formaldehyde (2%) and glutaraldehyde (0.2%) for 5 min. In sequence, the cells were washed 3× PBS and incubated with the X-gal staining solution (X-gal 1 mg/mL; citrate–phosphate buffer 40 mM, pH 6.0; NaCl 150 mM; MgCl_2_ 2 mM; C_6_N_6_FeK_4_ 5 mM; C_6_N_6_FeK_3_ 5 mM) for 18 h in a dry incubator at 37 °C without CO_2_. The next day, cells were washed 3× with PBS and stained with DAPI (500 nM) at room temperature for 5 min in the absence of light. Subsequently, the β-Gal-positive cells were observed under a light microscope (Nikon Eclipse TS100, Tokyo, Japan) and analyzed with ImageJ^®^ software (version 1.54p). At least 300 cells were counted per well, and the percentage of senescence was calculated by the number of positive cells divided by the total number of cells.

### 4.6. Cell Viability

The colorimetric MTT (3-(4,5-dimethylthiazol-2-yl)-2,5-diphenyltetrazolium bromide) (Sigma-Aldrich, Saint Louis, MO, USA) tetrazolium reduction assay was used to evaluate cellular viability as previously described [50]. In brief, RAECs were cultured in a 96-well plate (2 × 10^4^ cells/well) for 24 h at 37 °C and 5% CO_2_ incubator. After this period, cells were pre-treated with different concentrations of terpenes (10^−9^, 10^−8^, 10^−7^, 10^−6^ M) in the presence and absence of D-galactose for 48 h at 37 °C in a humidified 5% CO_2_ condition. Then, MTT dissolved in PBS (pH 7.4) was added to each well (10 µL) to achieve a concentration of 0.45 mg/mL and incubated at 37 °C for 3 h. Subsequently, the cell culture medium was carefully replaced with 100 µL of solubilization solution to dissolve the formazan crystals. The cells were gently mixed to ensure complete solubilization, and the absorbance was recorded at 490 nm on a microplate reader (BioTek Instruments, Synergy HT, Winooski, VT, USA). Cell-free wells containing only medium were used for background subtraction. RAEC viability was expressed as a percentage of the control group.

### 4.7. ROS Measurements

Superoxide anions (O2•−) in RAECs were measured using the dihydroethidium (DHE) probe (Sigma-Aldrich, St. Louis, MO, USA) as a marker of oxidative stress, as previously described [51]. Briefly, cells were washed 3 times with PBS and then incubated with DHE (5 μM) at 37 °C for 40 min. After this period, cells were washed 3 times with PBS and immediately photographed using a Nikon^®^ Eclipse TS100 fluorescence microscope (Tokyo, Japan). Quantification of fluorescence intensity was performed using ImageJ^®^ software (version 1.54p) and normalized to the control group.

### 4.8. Statistical Analysis

Values were expressed as mean ± standard error of the mean (S.E.M). Statistical analysis was performed using one-way ANOVA following the Tukey post-test in the GraphPad Prism© software, version 8.0 (GraphPad Software Inc., La Jolla, CA, USA). Values of *p* < 0.05 were considered statistically significant.

## 5. Conclusions

In conclusion, the present study identifies terpenes that modulate senescence-associated phenotypes in D-galactose-treated endothelial cells. Among them, citral, terpinolene, and farnesol emerged as promising candidates for reducing stress and improving cell viability, whereas 1*R*-(-)-myrtenol and *trans*-caryophyllene warrant additional study to determine whether their preferential effects in D-galactose-treated cells reflect senolytic activity. These findings support further mechanistic studies to define the senescence-related actions of these compounds and their potential relevance for natural-product-based interventions targeting endothelial senescence.

## Figures and Tables

**Table 1:** SA-β-gal evaluation after 48 h of treatment with 20 different terpenes in an endothelial senescence model accelerated by D-galactose.

Terpenes	Ctl	Dgal 20 g/L	$\mathrm{Dgal}\;+\;10^{-9}$ M	$\mathrm{Dgal}\;+\;10^{-8}$ M	$\mathrm{Dgal}\;+\;10^{-7}$ M	$\mathrm{Dgal}\;+\;10^{-6}$ M
geraniol	15.07 ± 0.80	29.96 ± 0.40 *	28.00 ± 0.59 *	28.97 ± 2.20 *	23.61 ± 0.34 *#	22.39 ± 0.61 *#
citral	14.93 ± 1.24	29.94 ± 0.56 *	20.08 ± 1.86 #	26.77 ± 1.86 *	26.82 ± 1.23 *	28.27 ± 1.10 *
linalool	14.93 ± 1.24	29.94 ± 0.56 *	31.09 ± 1.11 *	24.65 ± 0.95 *#	25.51 ± 0.71 *#	23.98 ± 0.64 *#
linalyl acetate	16.81 ± 0.44	29.96 ± 0.40 *	30.39 ± 1.42 *	27.86 ± 1.43 *	26.21 ± 2.03 *	25.39 ± 1.59 *
β-citronellol	18.82 ± 0.68	36.67 ± 0.67 *	32.37 ± 1.42 *	32.41 ± 0.38 *	30.19 ± 0.89 *#	27.49 ± 1.55 *#
citronellal	12.21 ± 1.80	21.86 ± 1.41 *	14.64 ± 1.19 #	11.40 ± 1.20 #	12.46 ± 0.99 #	13.41 ± 1.41 #
α-terpineol	21.05 ± 0.37	39.48 ± 0.56 *	26.04 ± 1.77 *#	30.26 ± 2.22 #	26.41 ± 1.20 *#	31.39 ± 1.09 #
carvacrol	17.93 ± 0.42	31.67 ± 0.99 *	26.36 ± 1.03 *#	25.30 ± 1.36 *#	23.52 ± 0.54 *#	22.46 ± 0.78 *#
thymol	19.02 ± 0.37	32.17 ± 0.73 *	27.09 ± 1.36 *	25.91 ± 1.26 *#	19.53 ± 1.36 #	21.01 ± 2.08 #
ρ-cymene	12.30 ± 0.75	23.75 ± 1.82 *	20.58 ± 1.58 *	20.24 ± 1.40 *	17.27 ± 1.29 #	13.71 ± 1.00 #
terpinolene	16.81 ± 0.44	29.96 ± 0.40 *	28.84 ± 0.91 *	28.18 ± 1.41 *	22.23 ± 0.61 *#	18.46 ± 0.62 #
(-)-carveol	11.28 ± 0.83	22.28 ± 0.35 *	22.98 ± 1.27 *	18.06 ± 0.65 *	21.46 ± 0.43 *	20.26 ± 2.22 *
(-)-terpinen-4-ol	11.28 ± 0.83	22.13 ± 0.27 *	14.06 ± 1.59 #	12.47 ± 1.04 #	11.16 ± 1.50 #	11.79 ± 0.54 #
(+)-α-pinene	11.44 ± 0.70	22.32 ± 1.37 *	19.53 ± 2.01 *	19.03 ± 1.32 *	15.49 ± 0.98 #	15.51 ± 1.71 #
(-)-borneol	11.44 ± 0.70	22.32 ± 1.37 *	15.55 ± 1.27 #	14.19 ± 1.49 #	14.65 ± 0.78 #	12.25 ± 2.10 #
1*R*-(-)-myrtenol	12.30 ± 0.75	23.75 ± 1.82 *	18.35 ± 1.05	21.31 ± 1.06 *	20.86 ± 1.86 *	16.76 ± 0.42 #
farnesol	12.21 ± 1.80	21.86 ± 1.41 *	17.47 ± 0.23	17.30 ± 1.12	14.90 ± 0.97 #	10.41 ± 0.92 #
nerolidol	12.21 ± 1.80	21.86 ± 1.41 *	18.14 ± 1.79	15.27 ± 1.25	13.28 ± 2.24 #	16.08 ± 1.02
α-bisabolol	12.30 ± 0.75	23.75 ± 1.82 *	19.20 ± 1.95 *	16.73 ± 1.50 #	16.32 ± 0.51 #	16.54 ± 1.11 #
*trans*- caryophyllene	12.21 ± 1.80	21.86 ± 1.41 *	19.36 ± 0.55 *	16.45 ± 1.35	12.88 ± 0.58 #	13.84 ± 0.79 #

Terpenes concentrations are expressed in mol·L^−1^ (M). Each test represents the mean ± SEM (*n* = 3). Ctl = control; Dgal = D-galactose. * *p* < 0.05 vs. control; # *p* < 0.05 vs. Dgal 20 g/L.

**Table 2:** Cell viability evaluation after 48 h of treatment with 20 different terpenes in a basal condition in endothelial cells.

Terpenes	Ctl	$10^{-9}$ M	$10^{-8}$ M	$10^{-7}$ M	$10^{-6}$ M
geraniol	100 ± 3.42	98.64 ± 2.35	99.73 ± 2.98	98.10 ± 4.89	98.64 ± 2.70
citral	100 ± 3.57	97.79 ± 1.67	84.65 ± 3.80 *	83.68 ± 2.04 *	81.05 ± 4.72 *
linalool	100 ± 1.84	98.74 ± 2.33	100 ± 3.88	90.54 ± 1.36	90.70 ± 0.74
linalyl acetate	100 ± 1.84	94.72 ± 1.86	92.80 ± 2.20	91.62 ± 3.21	89.61 ± 2.76 *
β-citronellol	100 ± 2.23	98.42 ± 4.07	102.99 ± 3.51	99.34 ± 3.05	96.02 ± 3.97
citronellal	100 ± 1.09	98.38 ± 3.22	99.07 ± 2.60	99.07 ± 3.49	99.81 ± 2.93
α-terpineol	100 ± 1.45	98.98 ± 1.67	99.43 ± 4.82	99.74 ± 5.90	93.10 ± 3.02
carvacrol	100 ± 5.53	98.99 ± 2.78	97.32± 1.33	98.27 ± 2.58	99.72 ± 5.56
thymol	100 ± 1.63	99.15 ± 6.08	96.96 ± 5.03	95.44 ± 7.92	93.52 ± 8.34
ρ-cymene	100 ± 2.16	95.53 ± 2.35	96.24 ± 5.80	97.56 ± 4.48	93.09 ± 4.20
terpinolene	100 ± 5.18	97.63 ± 3.32	97.48 ± 1.21	98.07 ± 3.77	94.22 ± 3.06
(-)-carveol	100 ± 3.91	102.27 ± 2.63	98.90 ± 5.36	86.02 ± 2.16	84.30 ± 2.75 *
(-)-terpinen-4-ol	100 ± 1.63	102.23 ± 5.43	96.07 ± 4.15	90.34 ± 1.47	85.46 ± 1.77 *
(+)-α-pinene	100 ± 3.53	92.59 ± 2.45	87.56 ± 2.97	89.70 ± 2.35	85.56 ± 4.90 *
(-)-borneol	100 ± 2.42	98.57 ± 3.82	104.37 ± 7.00	99.29 ± 6.87	98.41 ± 3.86
1*R*-(-)-myrtenol	100 ± 2.14	96.84 ± 2.06	101.86 ± 4.36	98.79 ± 7.81	95.87 ± 7.89
farnesol	100 ± 2.23	95.11 ± 3.42	98.76 ± 2.30	106.30 ± 3.10	100.08 ± 4.14
nerolidol	100 ± 5.86	97.73 ± 13.62	94.11 ± 10.31	96.13 ± 8.75	93.01 ± 11.36
α-bisabolol	100 ± 2.23	94.86 ± 4.06	96.85 ± 5.40	94.03 ± 4.97	91.63 ± 3.74
*trans*- caryophyllene	100 ± 2.08	97.82 ± 5.78	94.01 ± 3.56	98.06 ± 4.39	97.33 ± 2.66

Terpenes concentrations are expressed in mol·L^−1^ (M). Each test represents the mean ± SEM (*n* = 6). Ctl = control. * *p* < 0.05 vs. control.

**Table 3:** Cell viability evaluation after 48 h of treatment with 20 different terpenes in an endothelial senescence model accelerated by D-galactose.

Terpenes	Ctl	Dgal 20 g/L	$\mathrm{Dgal}\;+\;10^{-9}$ M	$\mathrm{Dgal}\;+\;10^{-8}$ M	$\mathrm{Dgal}\;+\;10^{-7}$ M	$\mathrm{Dgal}\;+\;10^{-6}$ M
geraniol	100 ± 3.85	76.12 ± 2.95 *	99.34 ± 4.91 #	99.61 ± 7.12 #	87.14 ± 4.57	85.70 ± 1.86
citral	100 ± 2.55	83.81 ± 2.72 *	96.84 ± 0.99 #	75.86 ± 1.72 *	78.35 ± 1.13 *	78.64 ± 3.23 *
linalool	100 ± 3.28	79.88 ± 3.55 *	89.40 ± 2.41	90.83 ± 3.77	86.43 ± 1.73 *	78.45 ± 3.48 *
linalyl acetate	100 ± 4.68	84.00 ± 2.52 *	83.66 ± 7.98 *	78.59 ± 5.84 *	76.60 ± 6.90 *	67.88 ± 4.49 *
β-citronellol	100 ± 3.28	79.88 ± 3.55 *	82.74 ± 6.27 *	81.44 ± 2.61 *	92.98 ± 3.02	84.88 ± 4.20
citronellal	100 ± 3.28	79.88 ± 3.55 *	84.05 ± 1.47 *	92.26 ± 3.41	79.88 ± 2.25 *	86.67 ± 3.61 *
α-terpineol	100 ± 3.85	76.12 ± 2,95 *	99.61 ± 4,43 #	95.80 ± 7.12 #	82.81 ± 3.42	92.65 ± 3.88
carvacrol	100 ± 2.32	78.27 ± 2.09 *	90.99 ± 2.95	82.55 ± 1.42 *	82.43 ± 5.91 *	87.61 ± 0.67
thymol	100 ± 2.77	74.13 ± 5.09 *	75.22 ± 3.71 *	77.27 ± 5.67 *	74.78 ± 4.29 *	74.78 ± 2.30 *
ρ-cymene	100 ± 2.32	78.27 ± 2.09 *	76.69 ± 5.77 *	83.90 ± 1.92 *	89.86 ± 1.22	90.77 ± 1.58
terpinolene	100 ± 3.85	76.12 ± 2.95 *	99.74 ± 3.74 #	92.91 ± 4.79 #	95.80 ± 3.00 #	91.86 ± 2.09 #
(-)-carveol	100 ± 2.77	74.13 ± 5.09 *	76.62 ± 4.81 *	76.73 ± 3.76 *	75.97 ± 4.70 *	68.40 ± 2.62 *
(-)-terpinen-4-ol	100 ± 1.05	80.73 ± 3.05 *	85.45 ± 2.53	74.28 ± 3.95 *	74.18 ± 6.32 *	74.95 ± 5.08 *
(+)-α-pinene	100 ± 2.37	83.33 ± 0.54 *	87.59 ± 2.98	88.68 ± 4.84	88.04 ± 4.81	76.00 ± 4.41 *
(-)-borneol	100 ± 2.32	78.27 ± 2.09 *	85.25 ± 3.59 *	83.90 ± 5.15 *	80.63 ± 3.25 *	81.87 ± 2.47 *
1*R*-(-)-myrtenol	100 ± 2.37	83.33 ± 0.54 *	82.61 ± 2.87 *	72.28 ± 4.11 *	74.82 ± 2.45 *	65.85 ± 3.26 *#
farnesol	100 ± 2.86	81.33 ± 1.34 *	82.29 ± 2.60 *	90.19 ± 2.25	96.76 ± 2.91 #	85.62 ± 2.64 *
nerolidol	100 ± 3.94	83.73 ± 1.38 *	86.76 ± 4.52	83.24 ± 2.89 *	79.31 ± 3.98 *	81.67 ± 3.52 *
α-bisabolol	100 ± 2.43	82.00 ± 0.53 *	77.72 ± 1.58 *	84.94 ± 2.96 *	82.98 ± 2.22 *	87.79 ± 2.80 *
*trans*- caryophyllene	100 ± 2.86	81.33 ± 1.34 *	82.29 ± 4.23 *	82.57 ± 2.72 *	85.14 ± 1.63 *	65.71 ± 3.96 *#

Terpenes concentrations are expressed in mol·L^−1^ (M). Each test represents the mean ± SEM (*n* = 3). Ctl = control; Dgal = D-galactose. * *p* < 0.05 vs. control; # *p* < 0.05 vs. Dgal 20 g/L.

**Table 4:** Superoxide anion evaluation after 48 h of treatment with 20 different terpenes in an endothelial senescence model accelerated by D-galactose.

Terpenes	Ctl	Dgal 20 g/L	$\mathrm{Dgal}\;+\;10^{-9}$ M	$\mathrm{Dgal}\;+\;10^{-8}$ M	$\mathrm{Dgal}\;+\;10^{-7}$ M	$\mathrm{Dgal}+10^{-6}$
geraniol	100 ± 0.39	111.83 ± 0.91 *	110.58 ± 1.08 *	110.90 ± 1.65 *	104.30 ± 0.68 #	101.28 ± 0.30 #
citral	100 ± 0.06	114.35 ± 0.92 *	99.18 ± 0.57 #	98.81 ± 0.24 #	100.50 ± 0.48 #	96.30 ± 1.01 #
linalool	100 ± 0.38	117.60 ± 0.94 *	108.22 ± 1.64 *#	102.62 ± 1.17 #	102.29 ± 1.22 #	103.05 ± 0.35 #
linalyl acetate	100 ± 1.97	114.97 ± 0.50 *	114.38 ± 0.41 *	101.13 ± 0.27 #	104.72 ± 2.64 #	111.66 ± 1.86 *
β-citronellol	100 ± 2.03	117.77 ± 2.61 *	110.33 ± 1.66	105.40 ± 1.53 #	100.89 ± 2.63 #	102.32 ± 2.36 #
citronellal	100 ± 2.49	119.46 ± 1.89 *	112.24 ± 1.93 *	102.55 ± 2.95 #	102.00 ± 1.20 #	100.47 ± 1.67 #
α-terpineol	100 ± 0.39	110.90 ± 0.88 *	110.37 ± 0.81 *	108.22 ± 1.77 *	106.02 ± 2.69	101.63 ± 0.35 #
carvacrol	100 ± 1.27	119.18 ± 2.80 *	115.54 ± 1.09 *	107.56 ± 1.48 #	117.04 ± 3.49 *	124.84 ± 1.69 *
thymol	100 ± 0.72	120.64 ± 2.03 *	106.11 ± 0.87 #	106.04 ± 1.33 #	107.43 ± 4.31 #	110.16 ± 2.59
ρ-cymene	100 ± 3.19	117.89 ± 2.77 *	109.41 ± 3.75	116.47 ± 4.24	109.81 ± 3.57	107.46 ± 4.70
terpinolene	100 ± 2.49	119.46 ± 1.89 *	111.44 ± 3.18	111.59 ± 3.15	101.28 ± 1.49 #	103.26 ± 2.48 #
(-)-carveol	100 ± 0.72	120.64 ± 2.03 *	103.21 ± 2.18 #	106.89 ± 0.87 #	103.51 ± 0.67 #	102.45 ± 3.53 #
(-)-terpinen-4-ol	100 ± 0.20	111.61 ± 0.25 *	107.80 ± 0.43 *	102.61 ± 0.40 #	103.39 ± 0.69 #	102.83 ± 2.03 #
(+)-α-pinene	100 ± 0.20	111.61 ± 0.25 *	108.74 ± 0.86 *	109.32 ± 1.06 *	101.84 ± 1.50 #	100.81 ± 1.00 #
(-)-borneol	100 ± 1.27	119.18 ± 2.80 *	119.73 ± 1.61 *	116.63 ± 2.62 *	110.48 ± 3.42	102.06 ± 1.16 #
1*R*-(-)-myrtenol	100 ± 2.12	126.67 ± 1.81 *	121.26 ± 3.82 *	131.22 ± 2.11 *	124.08 ± 2.07 *	123.28 ± 3.58 *
farnesol	100 ± 0.48	116.19 ± 0.49 *	115.79 ± 1.20 *	113.26 ± 1.70 *	106.18 ± 2.44 #	100.94 ± 0.96 #
nerolidol	100 ± 1.14	127.30 ± 0.56 *	119.28 ± 1.90 *#	114.54 ± 1.21 *#	112.91 ± 1.39 *#	105.64 ± 0.91 #
α-bisabolol	100 ± 0.48	116.19 ± 0.49 *	91.86 ± 1.20 #	90.55 ± 2.34 *#	92.46 ± 2.83 #	89.98 ± 2.26 *#
*trans*- caryophyllene	100 ± 2.12	122.98 ± 1.40 *	119.54 ± 1.19 *	97.14 ± 1.62 #	105.12 ± 2.26 #	108.66 ± 3.00 #

Terpenes concentrations are expressed in mol·L^−1^ (M). Each test represents the mean ± SEM (*n* = 3). Ctl = control; Dgal = D-galactose. * *p* < 0.05 vs.control; # *p* < 0.05 vs. Dgal 20 g/L.

## Data Availability

Data are available from the corresponding author upon request.
